# 

**Published:** 1890-01-18

**Authors:** 


SATURDAY, JANUARY 18th, 1890.

**Do Working Men Think?** - 239
**Words of Consolation**.—Gifts to Christ - 240
**The Doctor's Pulpit.**—Medical and Common Morality - 241
**Influenza** - 242
**Calendrier de Sante, 1890** - 242
**The Abuse of Hospitals.** By JOHN J. WEAVER, House Surgeon, Southport Infirmary - 243
**Pauperism in London.**—II. - 244
**Everybody's Page** - 245
**Notes and News** - 246
**Annotations.**—Human Development in Africa—Dr. Underwood's Opinions — Gentlemen "Pelicans" and "Low Organisms" - 248
**A Novelty in Disinfectants** - 251
**The Practitioner's Outlook and Retrospect:**
MEDICINE—Hysterical Amaurosis—Hepatic Abscess - 249
SURGERY—Rhinitis—Trephining for Cerebral Tumour Knee-Joint Disease - 249, 250
THERAPEUTICS—Incompatibles - 250
NEW DRUGS, APPLIANCES, AND THINGS MEDICAL—Stern's Pumiline Preparations—Sciandra's Preserved Natural Pulque - 250
**The Hospital Workshop.** XX.—St. Luke's Hospital for the Insane - 252
**Destitute Workmen's Aid Society** - 253
**Fallow Crops.**—The Hospital Sunday Fund and Institution for the Incurables - 254
**Scraps and Gleanings** - 254

**EXTRA SUPPLEMENT—THE NURSING MIRROR.**

**En Passant.**—Widely Read—Nursing at Edinburgh—South Hants Infirmary—Short Items—The Nurses Casebook—Grumblers—Nursing the Lepers—Their Little Best - lxv
**Mr. Trantum's Hobby** - lxvi
**The Nurses' Bookshelf** - lxvi
**Keeping Christmas** - lxvii
**Everybody's Opinion.**—Overworked Nurses - lxviii
**Presentations—Appointments** - lxviii
**Vacancies—Notices** - lxviii
**Amusements and Relaxation** - lxviii

